# Absorption and scattering properties of carbon nanohorn-based nanofluids for direct sunlight absorbers

**DOI:** 10.1186/1556-276X-6-282

**Published:** 2011-04-04

**Authors:** Luca Mercatelli, Elisa Sani, Giovanni Zaccanti, Fabrizio Martelli, Paola Di Ninni, Simona Barison, Cesare Pagura, Filippo Agresti, David Jafrancesco

**Affiliations:** 1 National Institute of Optics, National Council of Research, Firenze, 50125, Italy; 2 Department of Physics and Astronomy, University of Firenze, Sesto Fiorentino, 50019, Italy; 3 Institute for Energetics and Interphases, National Council of Research, Padova, 35127, Italy

## Abstract

In the present work, we investigated the scattering and spectrally resolved absorption properties of nanofluids consisting in aqueous and glycol suspensions of single-wall carbon nanohorns. The characteristics of these nanofluids were evaluated in view of their use as sunlight absorber fluids in a solar device. The observed nanoparticle-induced differences in optical properties appeared promising, leading to a considerably higher sunlight absorption with respect to the pure base fluids. Scattered light was found to be not more than about 5% with respect to the total attenuation of light. Both these effects, together with the possible chemical functionalization of carbon nanohorns, make this new kind of nanofluids very interesting for increasing the overall efficiency of the sunlight exploiting device.

**PACS:**

78.40.Ri, 78.35.+c, 78.67.Bf, 88.40.fh, 88.40.fr, 81.05.U.

## Introduction

Heat exchange devices suffer a serious limitation due to the relatively low thermal conductivity of conventional fluids. Because of solid materials have typical thermal conductivities order-of-magnitude higher than that of liquids, the possibility to add some amount of solid particles to conventional heat transfer fluids has been widely investigated in the literature [1-4] since the pioneering works done in seventies [5,6]. Anyway, as the thermal conductivity enhancement is dependent on the surface area-to-volume ratio, the use of nanometer-sized, non-spherically shaped particles offers the largest advantage.

In the fields of the research and technology involving the exploitation of renewable energies, solar thermal collectors have gained an increasing role in the last years [7]. The so-called "thermal" sunlight exploiting devices are heat exchangers that transform the energy of solar radiation to internal energy of some exchange medium. Typically, solar energy is absorbed by black-surface tubes (typically, a black-painted or oxidized surface in tight thermal contact with the tubes) [7], but this configuration entails various limitations. Therefore, alternative concepts have been addressed like the use of black particles suspended in gases [8] or in liquids as both solar radiation absorbers and heat transfer medium. In fact, carbon particles in gases have been demonstrated to be very efficient for devices operating in the high temperature range [9], whereas collectors employing black liquids within a transparent tube result advantageous in the low and medium temperature working range [10]. Conventional black liquids are based on organic inks or Indian ink, but they show serious drawbacks because of the light-induced degradation and thermal degradation at the operating temperatures, instability of solutions during time and fouling of inks on the internal side of exposed surfaces.

For these reasons, the use of nanofluids in solar thermal collectors is being investigated. Different spherically shaped materials, such as aluminium [11] and copper [3] have been added to different base fluids and characterized in terms of their performances for improving heat transfer efficiency. Materials with higher surface area-to-volume ratio due to a markedly aspherical shape have been investigated as well, like multiwall [12-15] and single-wall [16] carbon nanotubes (CNTs). CNTs are characterized by themselves by a very high thermal conductivity [12,17,18] and, also when suspended in fluids, they have been demonstrated to be very promising to enhance the heat transfer within the fluid [14-16,19]. Carbon nanohorns (CNHs) are the latest to be discovered in the family of carbon-based nanostructured materials [20]. They have a large surface area and a large number of cavities [21], and therefore appear promising for a variety of applications, such as electrode material in fuel cells [22], gas storage material [23], and carrier vehicles for delivering therapeutic drugs, genes or proteins [24-26].

A single-wall carbon nanohorn (SWCNH) consists of a single layer of a graphene sheet wrapped into an irregular tubule with a variable diameter of generally 2-5 nm and a length of 30-50 nm; the tips of the nanohorns are cone-shaped with an average angle of about 20° [27-29], corresponding to five pentagonal carbon rings at the tip of the tubule. The SWNHs assemble to form roughly spherical aggregates of mainly three types, known as dahlias, buds and seeds [29], depending on the synthesis conditions [20,30]. Among these possible aggregation states, the dahlia-flowerlike morphology has emerged as an interesting material within the family of carbon nanostructures [31] because it can be produced in high quantities and with high purity [20,30,32]. In fact, the critical points that differentiate CNH from CNT are their high purity due to the absence of metal nanoparticles (typically Fe and Co) used to catalyze nanotube growth during their production, the heterogeneous surface structure due to their highly strained conical ends, and finally the aggregation in spherical superstructures, typically ranging between 50 and 100 nm. Moreover, the rough surface structure of CNH aggregates with minimum Van der Waals interactions between the superstructures gives rise to better dispersion of CNH in liquid media [33] and a much longer time stability of their suspensions. Moreover, a very important property in view of their potential use with respect to CNTs arises from the metal-free structure of nanohorns that makes their cytotoxicity negligible, as has been widely confirmed by experiments on mice and rats [34]. This makes the use of SWCNH very appealing in all applications where the handling by operators is needed or when accidental leakages into the environment are possible.

Up to now, among the physical properties of nanofluids, the ones which usually are evaluated in view of practical applications are thermal conductivity [1-6,11] and pool boiling heat transfer [35-37]. However, as nanofluids appear promising for thermal solar energy applications, it is also important to carry out investigation of their optical properties allowing to assess their potentialities as direct sunlight absorbers.

Very recently, aqueous suspensions of SWCNH have been studied in view of this possible use [38]. However, in solar thermal collectors, water is often replaced by glycols or water/glycol mixtures, to protect against freeze damage and/or to increase the temperature for high temperature solar collectors, since glycols have lower freezing point and higher boiling point with respect to water.

Therefore, in this article we comparatively evaluated the thermal stability and the spectral absorption properties of water-based and glycol-based SWCNH suspensions, in the perspective to use them as direct absorber fluid in a sunlight collecting device. In addition, to evaluate the relative weight of scattering processes over the overall light extinction phenomenon, the absorption and scattering coefficients of SWCNH aqueous suspensions were separately measured with the utilization of a simple and precise technique. This allowed to assess the reliability of the measured transmittance data. This extensive analysis of SWCNH nanofluids can optimize their use, providing useful information for the system dimensioning.

## Results and discussion

### Sample preparation and structural characterization

A patented method (Carbonium Srl) [39] was used to produce very pure SWCNHs. This method demonstrated excellent capability and, differently from other methods commonly used, could be easily scaled up for massive production [40].

SWCNHs were mechanically dispersed in water and ethylene glycol by a high pressure homogenizer. In the case of water dispersions, the use of a dispersant resulted necessary and the sodium *n*-dodecyl sulphate (SDS, 99%, Alfa Aesar) was demonstrated as the best dispersant for this kind of carbon nanostructure. With this procedure, long-term stability was assured to the dispersions, as demonstrated by long-term stability measurements performed for the water dispersions [41] and by size measurements performed in glycol dispersion 6 months after the preparation (Sani E et al, Potentialities of carbon nanohorn-based suspensions for solar thermal collectors, submitted ).

In view of their potential application in solar thermal collectors, a preliminary inspection of the dispersion stability at high temperature was performed. For this aim, we carried out 5 cycles of quick heating and cooling in a sealed vessel at various temperatures. As to the dispersions in water, for temperatures up to 120°C, a good stability was observed, while at higher temperatures some aggregation phenomena and a partial settling was detected. As to the glycol dispersions, considering the higher boiling point of the base fluid, a higher stability was observed up to 150°C, where only a weak aggregation started.

The result of these very preliminary high temperature stability studies shows that this is a critical facet. Investigations are in progress to improve the suspension stability at higher temperature for solar thermal applications, while in case of a standard solar water heater the observed stability seems already sufficient. It should be emphasized that these preliminary measurements have been performed for SWCNH suspensions without optimization of nanoparticle concentration or of nanoparticle/surfactant concentration ratio. It seems reasonable to expect that an optimized surfactant amount will considerably improve the nanofluid high temperature stability.

### Scattering and absorption coefficients measurements

Scattering and absorption are connected each other by the extinction phenomenon. The Lambert-Beer law gives the relationship between the light extinction after a path length *r *within a generic medium showing both absorption and scattering effects:(1)

where *I*_0 _is the intensity of the input light, *I*(*r*) is the unscattered light intensity (ballistic component) measured after the distance *r *and μ_ext _is the extinction coefficient, which is given by the sum of the absorption μ_a _and scattering μ_s _coefficients:(2)

The relative contribution of scattering to extinction is given by the albedo ω:(3)

The experimental measurement of both absorption and scattering coefficients needs some additional definitions and the use of the diffusion equation. The details can be found in [42] and references therein. Here we will summarize only the basic facts.

The fluence rate Φ in a homogeneous and infinite diffusing medium at a specific distance *r *from a point-like CW source [42] emitting an unit power is given by:(4)

where  is the reduced scattering coefficient of the medium ( = μ_s _(1 - *g*) with *g *anisotropy factor of the scattering function [42]) and μ_eff _=(3 μ_a _)^1/2 ^is the effective attenuation coefficient.

Therefore, by means of multidistance fluence measurements, it is possible to obtain, with a linear fit, the effective attenuation coefficient μ_eff _of the medium. In fact, if we apply the natural logarithm of both sides of Equation 4:(5)

we can obtain μ_eff _as the slope of this linear relationship.

However, it is apparent that a single multidistance measurement is not sufficient to obtain both μ_a _and . To assess the values of both coefficients, it is necessary to repeat the measurement by changing only one of them in a controlled way, while the other remains unaltered. The approach we used to uncouple the two coefficients was:

1. preparing a suspension with a previously calibrated diffusive medium having both high scattering and small absorption, in our case Intralipid-20% (Fresenius Kabi, Uppsala, Sweden);

2. then modifying the absorption properties of the overall suspension introducing very small quantities of SWCNH suspension that does not significantly modify the scattering property of the resulting fluid (nanofluid additions lower than 0.1%);

In this way, we were able to modify only the absorption properties of the suspension, without modifications of the scattering behaviour.

If the medium of interest is obtained from a dilution of a given suspension, the absorption and scattering coefficients are connected to the respective values for the original non-diluted suspension by the relationship:(6)

where μ generically labels the coefficient under investigation, ε is its respective value for the non-diluted suspension and ρ is the concentration of the absorbing/scattering medium within the resulting suspension.

Once a specific quantity of absorber is introduced in the calibrated suspension, the expression of effective attenuation coefficient as a function of the added concentration of SWCNH suspension ρ_nh _becomes [43]:(7)

where μ_a0 _and  are the known absorption and reduced scattering coefficient of the calibrated Intralipid suspension, and ε_anh _the absorption coefficient of the original suspension of SWCNHs, before to dilute it in Intralipid suspension (as for the investigated concentration values of the original SWCNH suspension, the whole method was repeated for 5 samples with original concentrations ranging from 0.006 to 0.1 g/l).

The relationship between  and ρ_nh _is linear (Equation 7). Therefore, it is possible to obtain the absorption coefficient ε_anh _as the slope divided by .

On the other hand, the extinction coefficient can be obtained by a simple transmittance measurement. The explicit expression for the ballistic light transmitted through a cell of length *D *can be obtained by substituting the expressions (2) and (6) in Equation 1:(8)

where ε_snh _is the scattering coefficient of non-diluted suspension of SWCNHs. Again a linear equation can be used to solve for the extinction coefficient once the measurement is repeated for increasing quantities of SWCNHs [44]:(9)

The experimental setup for fluence rate measurements in the infinite medium is composed by a chopped He-Ne laser (632.8 nm) which is introduced in the medium by means of an optical fibre whose extremity radiates isotropically in the medium. A second identical optical fibre mounted on a computer-controlled translator receives the radiation in correspondence of a set of fixed distances from the emitting fibre [43]. The signal received by a photomultiplier coupled to the fibre is acquired by a lock-in amplifier.

The setup for transmittance measurement is composed by a calibrated length cell and by a chopped He-Ne laser. The light transmitted through the cell was measured with a photodiode and a lock-in amplifier. The experimental setup was similar to that of Ref. [43] with an acceptance angle of the detection system of 7 mrad. With this small acceptance angle the error on the extinction coefficient due to the unavoidable fraction of scattered received power was negligible, and the specific extinction coefficient has been obtained with an error smaller than 0.5%.

**Figure 1:**
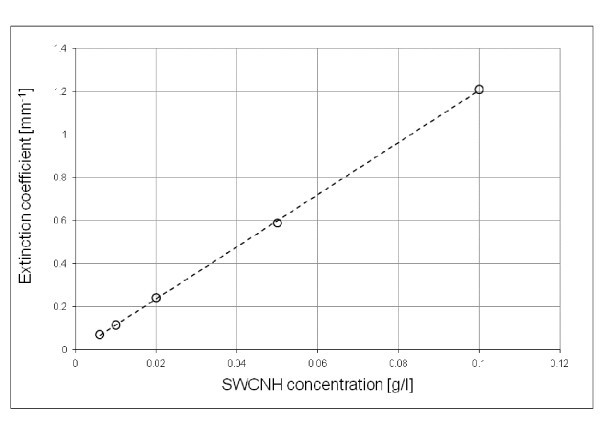
**Extinction coefficient as a function of SWCNH concentration, for the whole investigated concentration range**. The desired large range of optical densities was obtained applying the described dilution method for increasing the SWCNH original concentrations. For clarity reasons, only the points corresponding to the original concentrations are shown.

To evaluate the extinction coefficient and its linearity for a large range of SWCNH concentrations, the described dilution method was applied to various samples, i.e. for different starting SWCNH concentrations, as explained above. Each original concentration showed a linear behaviour of extinction coefficient versus diluted concentration. On the other hand, different original concentrations showed themselves an overall linear trend (Figure 1). According to the method, the slope of the straight line that best fits the results for each original concentration represents the specific extinction coefficient of SWCNHs at the laser wavelength, that results ε_enh _= 12.1 mm^-1 ^g/l and it is the same for all the investigated original concentrations and in the whole concentration range.

**Figure 2:**
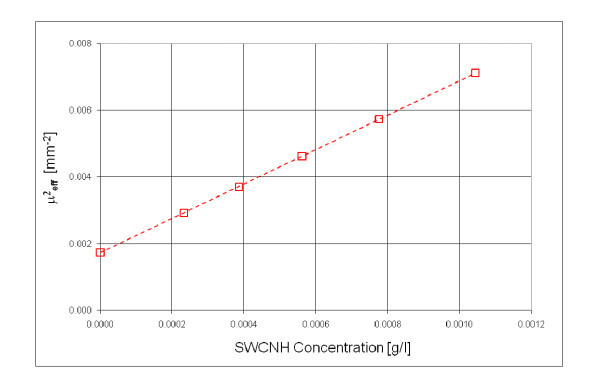
**Quadratic effective attenuation coefficient as a function of SWCNH concentration (the concentration of non-diluted suspension is 0.1 g/L)**.

Figure 2 shows the experimental values of the quadratic effective attenuation coefficient as a function of the SWCNH concentration. The absorption coefficient was calculated from the slope of the straight line that best fits the experimental results. The obtained absorption coefficient was ε_anh _= 11.4 mm^-1 ^g/l.

Therefore, the albedo of SWCNH suspension in water was only 0.05, confirming that the contribution of the scattered part to the total extinct light was very low. This value should be compared with those obtained in case of Indian ink aqueous suspensions, where the albedo was measured to be 0.15 [45]. This favourably compares SWCHN suspensions with respect to more conventional black fluids employed in solar collectors also for their minimum scattering contribution to the total extinct light.

The small fraction of scattered light allows to easily estimate the light absorption by SWCNH-based nanofluids by means of simple spectrophotometric measurements.

### Spectrophotometric measurements

Optical transmittance spectra at room temperature have been measured using a double-beam UV-VIS spectrophotometer (PerkinElmer Lambda900). The nanofluid is held in quartz cuvettes, with 10 mm beam path length. The investigated samples are listed in Table 1 and are labelled as X1...X5 for increasing SWCNH concentrations, with X = A for aqueous and X = G for glycol-based suspensions. For a more meaningful characterization of the SWCNH nanofluid, the transmittances of the pure base fluids (labelled as A0 and G0) were also measured for reference. The transmittance measurement was not possible for samples with concentrations higher than 0.06 g/l for glycol and 0.05 g/l for water suspensions because their transmitted light fell below the detection limit of our instrument. Anyway the optical properties we measured on more lightly concentrated samples can be easily scaled to higher concentrations if needed.

**Table 1:** Samples for spectrophotometric measurements

Label	SWCNH Concentration (g/L)
A0	No SWCNH

A1	0.005

A2	0.01

A3	0.02

A4	0.05

G0	No SWCNH

G1	0.005

G2	0.01

G3	0.02

G4	0.05

G5	0.06

**Figure 3:**
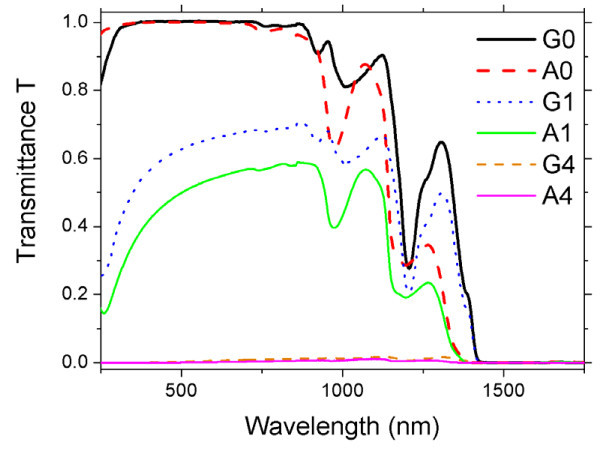
**Transmittance spectra of water and glycol-based nanofluids with the same SWCNH concentrations (0.005 g/L for G1 and A1 and 0.05 g/L for G4 and A4)**. Spectra of the pure base fluids are shown for comparison (G0 and A0).

The acquired transmittance spectra were corrected for the reflectance term. In Figure 3 we show the comparison of some transmittance spectra of pure fluids and nanofluids of both kinds with the same SWCNH concentrations.

From Figure 3 we can see that water and glycol show some spectral differences in their transmitting properties mainly for infrared wavelengths. SWCNHs produce a general decrease of the transmittance of the resulting nanofluid for almost all wavelengths and with a significant ultraviolet transmittance minimum due to nanoparticles. In general, glycol suspensions show a lower transmittance than water suspensions at fixed SWCNH concentration.

For a more significant assessment of the transmission/absorption properties of the nanofluid in view of the peculiar application in a sunlight collector, we evaluated the absorbed sunlight fraction *F*(*r*) as a function of the light path length r within the fluid. For this calculation, the fluid was considered still and cold (no convective mixing). We convoluted the Lambert-Beer law (which gives the light extinction within a medium) with the sunlight spectrum with air mass = 1.5 [46], and we calculated *F*(*r*) according to the equation:(10)

where α(λ) is the spectral absorption coefficient calculated from the transmittance spectra and *I*(λ) is the considered Sun spectrum.

The calculated values of *F*(*r*) for the various samples are tabulated in Table 2. As we can see, the sunlight absorption by pure base fluids is quite low, as *F*(*r*) remains lower than 40% for a light path as long as 10 cm. For this reason, usually absorber tubes that use water or water-glycol mixtures as heat transfer fluids are black-painted to increase the sunlight absorption [7]. Anyway with this configuration the contact between absorber and fluid is limited with consequent non-optimal thermal energy transfer. The addition of SWCNH considerably enhances the sunlight absorption, even for very low SWCNH concentrations. As we can see from Table 2, suspensions with only 0.005 g/l SWCNHs entail a light absorption of about 54% for water-based and about 43% for glycol-based fluids after the first centimetre of propagation, with a 96% (water suspension) and 89% (glycol suspension) absorption after 5 cm. If we compare this result with that of a 10-cm propagation within pure fluids (37 and 33% for water and glycol, respectively), the advantage given by SWCNHs is immediately evident.

**Table 2:** Stored energy fraction *F*(*r*) for the various samples for different sunlight path lengths

SWCNH concentration (g/l)	Sample label (base fluid)	*F*(*r*) *x *= 1.0 cm (%)	*F*(*r*) *x *= 2.5 cm (%)	*F*(*r*) *x *= 5.0 cm (%)	*F*(*r*) *x *= 10.0 cm (%)
Pure base fluid	A0 (water)	20.5	26.7	31.6	36.9

Pure base fluid	G0 (glycol)	17.4	23.0	28.0	33.4

0.005	A1 (water)	54.4	82.0	95.7	99.8

0.005	G1 (glycol)	43.2	70.1	89.3	98.5

0.01	A2 (water)	72.9	95.0	99.6	100

0.01	G2 (glycol)	68.0	92.7	99.3	100

0.02	A3 (water)	90.9	99.6	100	

0.02	G3 (glycol)	88.3	99.4	100	

0.05	A4 (water)	99.6	100		

0.05	G4 (glycol)	99.1	100		

If the SWCNH concentration grows, the absorption within the fluid becomes more localized in the first layers. In fact, *F*(*r*) values are around 70% after 1 cm and more than 90% after 2.5 cm for 0.01 g/l concentration in both fluids, whereas for 0.02 g/l concentration, the absorption is around 90% after 1 cm and practically 100% for 2.5 cm. For the highest SWCNH concentration in Table 2 (0.05 g/l), the absorption is almost total within the first centimetre for both liquids.

This demonstrates the significant role of SWCNHs in considerably enhancing the sunlight absorption properties of the fluid.

As we can see, the absorption becomes more localized near the surface as the SWCNH concentration increases. Therefore, for a still fluid and in absence of any motion, a temperature gradient between the fluid lying near the surface and the fluid lying near the tube centre should be expected. Anyway, it should be emphasized that our simple model does not include any fluid motion or mixing. The aim of calculation in Equation 10 and Table 2 was to quantitatively evaluate the effect of SWCNHs on the absorption properties of the fluids, and not to show temperature distributions within the fluid. In fact, the still liquid is only an ideal approximation, as the real fluid will undergo both convective motions due to temperature gradients and a collective motion along the tube length due to external pumping. Both these effects, combined with the expected thermal conductivity enhancement due to SWCNHs similar to that observed in CNTs suspensions [16,19], will contribute to uniform the fluid temperature for an optimal heat exchange.

## Conclusions

This article reports on the characterization of SWCNH-based nanofluids for thermal solar applications. Preliminary thermal stability tests have been performed, showing that water-based suspensions are stable up to 120°C, while glycol-based suspensions are stable up to 150°C. An improvement of the thermal stability properties of the nanofluid should be expected by an optimization of SWCNH-surfactant concentration ratio.

As for the nanofluid optical properties, the described measurements show that nanohorn-based suspensions are very promising direct sunlight absorbers for thermal solar energy exploitation. We carried out both spectrally resolved and light scattering characterizations. We demonstrated that only small amount of radiation (~5% of the total extinction) was scattered by SWCNH particles and therefore the absorption effect was strongly prevailing. Moreover, the small scattering was also combined with an overall high absorption level at the sunlight emission wavelengths. Both these effects make SWCNHs-based nanofluids very appealing as direct absorbers in innovative solar collectors. The reported scattering and absorption properties can be used in simulations and nanofluid optimization.

## Abbreviations

CNHs: carbon nanohorns; CNTs: carbon nanotubes; SWCNH: single-wall carbon nanohorn; SDS: sodium *n*-dodecyl sulphate.

## Competing interests

The authors declare that they have no competing interests.

## Authors' contributions

LM, ES, DJ: carried out spectrophotometric characterization. GZ, FM, PDN: carried out optical scattering measurements. SB, CP, FA: carried out sample preparation and high-temperature stability study. All authors read and approved the final manuscript.
